# Prevalence of *Helicobacter pylori* infection in Sari Northern Iran; a population based study 

**Published:** 2019

**Authors:** Iradj Maleki, Masoumeh Mohammadpour, Niloufar Zarrinpour, Mohadeseh Khabazi, Reza Ali Mohammadpour

**Affiliations:** 1 *Associate professor of gastroenterology, Gut and Liver Research Center, Mazandaran University of Medical Sciences, Sari, Iran*; 2 *Medical Student, Ramsar International Campus, Mazandaran University of Medical Sciences, Sari, Iran*; 3 *Pathologist, Technical assistant of Health Center No.5 Laboratory, Sari, Iran*; 4 *Associate professor, Department of Biostatistics, Faculty of Health, Mazandaran University of Medical Sciences, Sari, Iran *

**Keywords:** *Helicobacter pylori*, Prevalence study, Epidemiology, Iran

## Abstract

**Aim:**

In this study prevalence rate of *Helicobacter* *pylori* and its associated factors have been investigated in urban and rural areas of Sari.

**Background:**

*Helicobacter* *pylori* has an important role in gastrointestinal diseases including peptic ulcer and gastric cancer. It is the most common infection in human population worldwide. Hence, the epidemiology of this infection in all parts of the world is of utmost importance.

**Methods:**

We conducted a cross-sectional study on 497 individuals ranging 15-65 years of age in Sari city and its surrounding rural residents. The sampling method was a cluster random sampling multi staged in stratified population by urban and rural areas. Questionnaires for personal and socio-economic data were filled. Blood samples were drawn and kept for analysis (IgG antibody ELISA for *Helicobacter pylori*). The data was analyzed by SPSS statistical software and Chi-square test and logistic regression were used.

**Results:**

The prevalence of helicobacter infection was 44.5% in the studied population. This prevalence was 41.3% and 47.8% in urban and rural areas, respectively. Just a significant association between the infection and the age of subjects was observed in multiple regression analysis (p=0.001). However, in univariate analysis the level of education was also significantly associated with *Helicobacter pylori* infection (p=0.015). No other variable was associated with the infection.

**Conclusion:**

The prevalence of helicobacter infection has dropped significantly in the region in comparison with the previous studies during the last 15 years.

## Introduction

*Helicobacter pylori* is a spiral Gram-negative bacillus that plays an important role in the pathogenesis of upper gastrointestinal disorders (1, 2). *Helicobacter* *pylori* is a common infection and it has been estimated that about half of humans' population have experienced infection with *Helicobacter* *pylori* (3, 4). Infection with this bacteria is an important etiologic factor of gastritis, peptic ulcer disease and gastric adenocarcinoma or gastric MALTOMA, although it remains asymptomatic in most cases (5). 

More than 80 percent of duodenal ulcers and more than 60 percent of gastric ulcers universally are related to *Helicobacter* *pylori* colonization (6). Frequency of *Helicobacter* *pylori* infection in adults has been reported approximately 30% in developed countries and up to 80% in most of developing countries. Prevalence of infection in young people in developing countries is more than developed countries (7).

Several studies have been done to clarify the prevalence of *Helicobacter pylori* infection in different regions of the world with various results in different parts of the globe (8, 9). According to various evaluations conducted in the world, factors such as economic-social class and education have been effective on the rate of prevalence and race has not been effective. 

Also defect in sanitation, unsafe drinking water, poor and unsanitary nutrition and living at crowded houses have been associated with this infection (7). A study has shown that prevalence rate of *Helicobacter* *pylori* infection was high (74.4%) in a region in the Middle East and increased with increasing age (10). This value has been different in various provinces in Iran according to economic, cultural and geographical conditions and dates performed.

 Mazandaran province is in north of Iran where is considered as a hotspot area for gastric adenocarcinoma and this cancer has been known as the most prevalent tumor in this region. *Helicobacter* *pylori* is a well-known carcinogen in the genesis of gastric cancer, so periodic evaluation of epidemiology of this micro-organism in this region has an obvious importance. 

Sari, the capital city of Mazandaran has a mixture population from all districts of the province due to centrality, geographical and administrative status and sampling from this city can be considered as an index of the whole province. Considering these data, we decided to evaluate the prevalence of *Helicobacter* *pylori* infection in urban and rural areas of Sari and also evaluate the associated factors related to this infection. 

## Methods

The study was intended to be conducted on 500 cases of residents in Sari city and its neighboring rural areas within the ages of 15 to 65 years old. The sampling method was a cluster random sampling multi staged in stratified population by urban and rural areas. In the first stage, four rural health care centers (with 15 villages) were selected in four directions: north, south, east and west of the rural areas of Sari and three urban health care centers. Then, in the second stage, the clusters were defined according to the list of households in each center, and the individuals were identified in proportion to the gender and age groups. After the required coordination with health care centers based on urban and rural households list, number of clusters with 20 families systematically random were selected and the cases were reached at their home addresses. Home addresses and phone numbers were registered in the center. The cases were informed by phone about the study by the health care workers in advance. The minimum sample size was determined with 95% confidence level as 246 and 245 people in urban and rural regions respectively based on previous study (11). 

Before collecting blood sample, a questionnaire was given to patients containing some personal questions including age, sex, job, smoking and some parameters of hygienic status and sampling was conducted after completing the form. To evaluate parameters about hygienic status questions were asked on the type of drinking water, sewage disposal, oral and dental hygiene and to evaluate social status other questions were asked about their home, number of family members and level of education. A 3 cc of venous blood was drawn and stored at refrigerator and transferred to the main laboratory center. Titer of IgG anti-Helicobacter pylori antibodies was measured (HP IgG ELISA kit of Pishtaz Teb Company). Sensitivity and specificity of ELISA kit reported respectively 97% and 91% by Pishtaz Teb Company (www.pishtazteb.com). The lgG titer was reported negative for values lower than 10 and positive for titers of 10 and higher. 

**Table 1:** Samples demographic situation, socio-economic factors and health status (n=497)

**Variables**		
Sex	FemaleMale	286(57.5)211(42.5)
Age groups (years old)	15-2526-3536-4546-5556-65	114(22.9)125(25.2)95(19.1)86(17.3)77(15.5)
Living area	UrbanRural	252(50.7)245(49.3)
job	Unemployed & housewivesStudent or collegianFarmerWorkerStaffSelf-employed	116(23.3) 48(9.7)24(4.8)26(5.2)123(24.7)160(32.2)
Education level	IlliterateElementaryMiddle & High schoolDiplomaPostgraduate	13(2.6)40(8)81(16.3)183(36.8)180(36.2)
History of	gastrointestinal diseases Gastrointestinal drugs Cigarette smoking and tobacco Oral diseases	91(18.3) 108(21.8) 57(11.5) 70(14.1)
Hygienic status	Access to sanitary drinking water	475(95.6)

**Table 2:** The prevalence rate in different studies

**Prevalence**	**Sample group**	**Location**	**Year**	**Author**
19.2%	7-10 year-old students	Sari	1999	A. Farhadi
61.6%	Referred to health centers	Kerman	2000	M. Zahedi
64.2%	General population	Sari	2002	F. Baba Mahmoudi
77.8%	Referred to Imam Reza Hospital Laboratory	Mashhad	2006	Z. Siadat
36.5%	The population over 10 years old	Kurdistan	2006	K. Yazdanpanah
15.3%	2-12 years old children admitted to Ghods hospital	Ghazvin	2006	A. Mahyar
76.4%	General population	Izeh	2008	A. Holaku
58.5%	General population	Arak	2011	A. Fani
74%	Referred to health centers	Sari	2013	M. Owrang

Prevalence of H*elicobacter pylori* infection was calculated and it was looked for associated factors in this group. The data was analyzed by SPSS statistical software (version 20) and Chi-square test and logistic regression were used. The p value of the lower than 0.05 was considered statistically significant. 

## Results

A total number of 497 cases were enrolled in the study. Demographic data of the study population is shown in table 1. *Helicobacter pylori* prevalence was found to be equal to 47.8% (95% CI (0.41, 0.53)) in rural regions in Sari city which is higher than urban area prevalence (41.3%)(95% CI (0.35, 0.47)) but this difference was not significant statistically (P=0.14). In urban region increase of infection had significant relation with age and in rural population it had significant relation with age and sex. Total prevalence was estimated 44.5% in both areas calculated together (95% CI (0.41, 0.48)). The sex difference was not shown in urban population and even not in merged data for all subjects.

**Figure 1:**
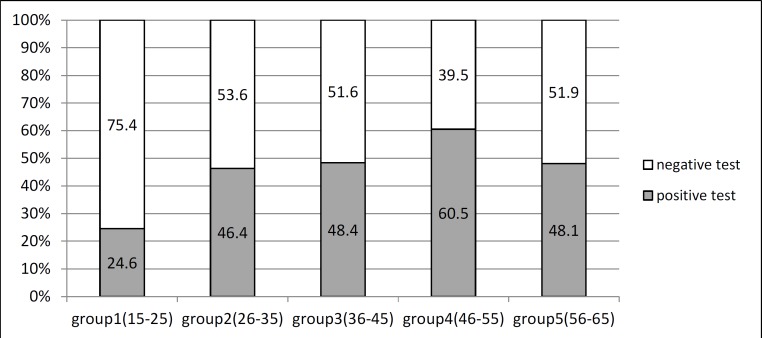
Frequency of serologic result for *Helicobacter pylori* in various age groups

Seropositivity for IgG antibodies against *Helicobacter pylori* in various age groups have been shown in figure 1. The maximum frequency of positive titer has been found in age group 4 (46 to 55 years old - 60.5%) and the lowest rate has been (24.6%) in age group 1 (15 to 25 years old). An obvious trend could be seen in the samples based on their age groups and there was a significant correlation between seropositivity for IgG antibody of *Helicobacter pylori* and the age groups (p=0.001).

There was no significant difference in seropositivity for *Helicobacter pylori* between women (41.3%) and in men (48.8%) in all data from rural and urban data merged together (P=0.09). Also there was no significant relation between this infection and various jobs (P=0.054). There was no significant difference between people born in Mazandaran (44.5%) and non-native people (44.4%) in frequency of positive titer (P=0.99).

**Figure 2:**
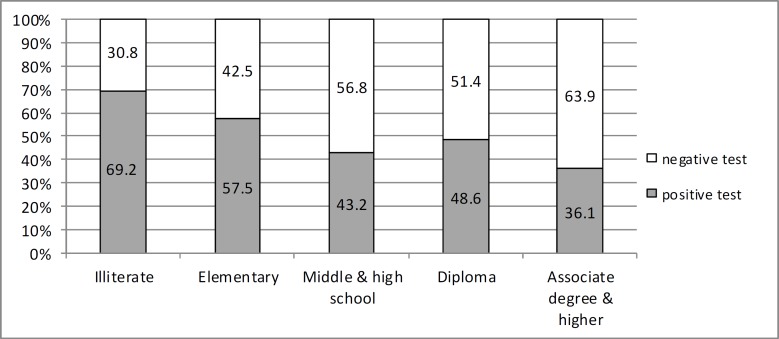
Frequency of serologic result for *Helicobacter pylori* in relation to education level

There was a significant correlation between *Helicobacter pylori* infection and the samples’ level of education in univariate analysis (P=0.01) (figure 2). It should be emphasized that the rate of infection is lower in the more educated samples. It is assumed that those who have the higher level of education at the time of the study, had a better socioeconomic status in their early childhood enabling them for higher education levels and also having better personal health in their childhood protecting them from acquiring the infection. There was no significant correlation between helicobacter infection and reported rates of gastrointestinal disease and use of gastrointestinal drugs (P=0.19) and (P=0.27) respectively. 

Although nonsmokers had a lower rate of helicobacter infection (43.4%) in comparison with smokers (52.6%), this difference was not significant statistically (P=0.18). 

By conducting logistic multi-variable regression, it was shown that the effect of age is significant (p=0.01) ((odds ratio=1.28 (CI95%; 1.06, 1.55)) and the other variables had no significant relation in predicting being positive or negative of the samples’ serological test. 

## Discussion

According to the present study the prevalence of *Helicobacter pylori* in urban and rural populations of Sari city were 41.3% and 47.8% respectively and the overall prevalence for both areas was 44.5%. Prevalence of *Helicobacter pylori* infection has dropped from 66.2% in a study about 15 years ago in the same population (11), which is equal to about 1.5% per year; however this drop could not be a linear one and the rate of this should have been dropped continuously. 

These two studies have been conducted in the same region and with comparable methodology as a population based study. Greatest differences are seen in younger age groups as it is expected, which shows the improvement of general health status and availability of piped water supply. During this time and with the eradication of *Helicobacter pylori* in the setting of peptic ulcer disease and other indications for eradication a subset of people has been potential reservoir for the spread of infection have been eliminated from the population too. 

Age and education level had significant correlation with the prevalence of *Helicobacter pylori* in univariate regression analysis but there was no significant correlation with other variables. The positive correlation with age has been shown universally in all studies performed in this region and all over the world; this effect has been known as “cohort effect” showing the importance of the general health status at the population in their first years of their life. In this study the lowest prevalence was in the youngest study group (15-25 years old) with a 24.6% seropositivity for *Helicobacter pylori*. In the other population based study in the same region performed 15 years earlier this figure was 28.5% in their youngest age group (under 10 years). In both studies the infection rose constantly with the increasing age and the difference of the infection can be shown significantly in all age groups.

In a study by Farhadi *et al*, (1999) (12) it was shown that the prevalence of *Helicobacter pylori* infection in 7 to 18 years old students in Sari city was 19.2%. This study was done in selected schools in the urban areas of Sari at a time near the study from Babamahmoodi, but the values for infection differ with near doubled value for the latter study which could be due to sample selection differences. The prevalence of *Helicobacter pylori* infection has been evaluated in many parts of Iran in the last 20 years with variable results (13, 14, 15, 16). These differences could be due different methodologies, different study population (population or clinic based), different age and sex groups and also real differences in geographic regions. A few studies have been introduced in the table 2.

Some principal differences have seen in some studies because of study designs. A study from Kerman (2000) with the overall prevalence of 61.6% showed no association with age in their samples. It should be mentioned that the study population was selected from a clinic and was not population based, hence a generalization cannot be done on their data in comparison with the population based studies (17). In most studies from all over the globe a logical positive correlation with increasing age have been reported. However, no significant correlation with sex was found in our study like other studies in Iran and region.

The level of education was conversely associated with the helicobacter pylori infection in our study. This has also been shown in some other local studies (12). This negative association is not etiologic in its base and concept. As most of people do get helicobacter in their childhood, a lot of time before they get educated, this reverse correlation may rather denote to the possible better socio-economic status of those who have the opportunity to reach better educational levels in their later life rather than the protective effect of education to *Helicobacter pylori* infection. However, one issue should be kept in mind considering the socio-economic status of the samples in these studies. In these studies, researchers consider the socio-economic status of the present time of the samples and not the one at the time when they were prone to the acquisition of the infection. Socio-economic status at these two times are not essentially the same; hence asking about the socio-economic status at childhood may be more beneficial in considering this variable in the etiology and pathogenesis of gastric *Helicobacter pylori* infection.

In a recent meta-analysis by Sayemiri *et al,* in 2014 on the available studies on the prevalence of *Helicobacter pylori *on 30 studies from all parts of Iran it has been shown that a variety of prevalence have been reported (19.2% to 74.3%) (18). The documentation of helicobacter infection was conducted by a variety of methods like serology, ELISA, PCR and even endoscopy and biopsy based on their study design. As it could be seen from their methodology the studied population have been very diverse. But by having more than 11000 samples, there was a decreasing trend in time in studies from 1993 to 2010. This concept is in favor with our findings in one region. It appears that the prevalence of *Helicobacter pylori* infection is decreasing in Iran, but the rate of this decrement could be different in various parts of the country. 

One of positive points of this research is evaluating all these variables in a logistic multivariable regression model that various variables in presence of each other have no considerable effect in explaining infection variance in different groups of the society. This point indicates that although separately changing hygienic behaviors and economic and social status in reduction of infection are emphasized in different researches, by changing each one of them no considerable change can be created in value of *Helicobacter pylori* infection so planning about providing hygienic and treatment services should be generalized for all groups of the society with each education level and income, both sexes and with each type of hygienic behavior and some innovations in theory and practice may be required in fighting against *Helicobacter pylori* and other human symbiotic bacteria with global massive prevalence.

We also have summarized a few pertinent studies of *Helicobacter *pylori infection performed in Iran which were performed during the last fifteen years. As the sampling and laboratory methods are different, no clear time trend can be shown in these studies.

Many researches have been conducted in different regions in the world such as research of Hoang in Vietnam with a prevalence of 74.6% (19), research of Sitas in Wales with a prevalence of 56.9 percent (20) and research of Asaka in Japan with a prevalence of 70 to 80 percent (21) and research of Graham et al in India with 79 percent (22). These mostly eastern world studies show higher prevalence of *Helicobacter pylori* in those populations in comparison with our study from northern Iran. In research of Smoak in the United States of America, prevalence of 43 percent in age group of 24 to 26 years old was higher than 17 to 18 years old with 24 percent (23) which is consistent with results of the present research and relation of prevalence value with age. But most recent studies from western world show lower rates of helicobacter infection in their population.

By this population based study we conclude that the prevalence of *Helicobacter pylori* infection is 44.5 percent in Sari, northern Iran which is lower than many regions in the world but high compared to developed countries. We have found that the prevalence of this infection has dropped significantly during the past 15 years. This infection is significantly correlated with age but other variables like sex, job, being native of Mazandaran, economic and social status of household are not correlated epidemiologic factors. We have not found any significance for type drinking water, mouth and tooth hygiene or smoking but titer of antibody is reduced with the change of scores of economic and hygienic levels
